# Supplementary material for: The Impact of the Synergistic Effect of Temperature and Air Pollutants on Chronic Lung Diseases in Subtropical Taiwan
Source: J Pers Med. 2021 Aug 21;11(8):819. doi: 10.3390/jpm11080819 (PMC8401456; doi:10.3390/jpm11080819)
Supplement: Supplementary file 1 [file jpm-11-00819-s001.zip › jpm-1306863-supplementary.pdf]

Table S1. Multiple comparison tests (post hoc analysis)

	Normal Spirometry(1)	Obstructive Impairment(2)	Restrictive Impairment(3)	Mixed Impairment(4)	Overall	2 vs 1	3 vs 1	4 vs 1
					P-value	P-value	P-value	P-value
n	1902	733	154	100				
Age (years), mean (SD)	49.51 (10.46)	50.56(10.68)	52.61 (11.02)	55.31 (11.25)	<0.0001	0.0664	0.0014	0.0000
30-39	438 (23.00)	149 (20.30)	26 (16.90)	15 (15.00)				
40-49	524 (27.50)	186 (25.40)	37 (24.00)	13 (13.00)				
40-59	585 (30.80)	231 (31.50)	49 (31.80)	26 (26.00)				
≥60	355 (18.70)	167 (22.80)	42 (27.30)	46 (46.00)	<0.0001	0.0115	0.0115	<0.0001
Only ≥60						0.5530	0.5530	0.0001
Sex, n (%)								
Male	903 (47.50)	322 (43.90)	66 (42.90)	32 (32.00)				
Female	999 (52.50)	411 (56.10)	88 (57.10)	68 (68.00)	0.0098	0.2039	0.2699	0.0087
Anthropometric parameter, mean (SD)								
Height (cm)	163.09 (8.32)	162.52 (8.05)	160.26 (7.57)	158.28 (7.80)	<0.0001	0.3271	0.0001	<0.0001
Weight (kg)	64.49 (12.22)	64.08 (11.60)	62.74 (12.94)	58.82 (10.67)	<0.0001	1.0000	0.2485	<0.0001
Body Adiposity Index	28.40 (3.92)	28.75 (3.79)	29.62 (4.38)	29.72 (3.79)	<0.0001	0.1140	0.0006	0.0031
Body Roundness Index	3.70 (1.12)	3.74 (1.08)	3.96 (1.38)	3.70 (1.20)	0.0475	1.0000	0.0163	1.0000
Biochemical data, mean (SD)								
Hematocrit (%)	44.32 (4.64)	43.86 (4.61)	44.13 (4.52)	43.10 (3.86)	0.0139	0.0674	1.0000	0.0291
Glycohemoglobin (%)	5.73 (0.71)	5.84 (0.90)	6.00 (1.13)	5.82 (0.73)	<0.0001	0.0033	0.0001	0.7781
Albumin (g/dL)	4.60 (0.24)	4.6 (0.24)	4.56 (0.25)	4.54 (0.24)	0.0241	1.0000	0.1620	0.0371
Diabetes mellitus type 2	77 (4.00)	43 (5.90)	12 (7.80)	6 (6.00)	0.0474	0.0935	0.0935	0.3433

Continuous variables of data were estimated by using the generalized linear regression model. Multiple comparison tests were calculated by using Bonferroni adjustment. Discrete variables of data were estimated by using the logistic regression model. Multiple comparison tests were calculated by using the MULTTEST procedure, a Bonferroni correction.

Table S2. Descriptive statistics of air pollutants and meteorological factors

	N	mean	SD	Median	Q1	Q3	Max	Min
Temperature (℃)	2889	24.33	0.77	24.41	23.96	24.83	26.36	21.46
Relative humidity (%)	2889	74.29	2.45	74.51	72.33	75.71	83.65	68.35
Rainfall (mm/day)	2889	0.22	0.05	0.21	0.19	0.25	0.38	0.06
PM_{10} (μg/m^3)	2889	68.07	17.06	70.54	58.16	79.41	117.62	27.79
$PM_{2.5}$ (μg/m^3)	2889	37.65	10.74	38.75	32.62	43.14	89.51	12.94
CO (ppm)	2889	0.45	0.18	0.46	0.36	0.48	1.06	0.15
NO (ppb)	2889	4.18	4.01	3.25	2.31	4.1	20.11	0.97
NO_2 (ppb)	2889	14.98	5.65	15.8	13.3	17.26	27.24	1.26
NO_x (ppb)	2889	19.14	8.93	19.23	16.24	21.05	47.01	2.24
O_3 (ppb)	2889	30.91	3.86	30.49	28.28	33.21	43.7	21
SO_2 (ppb)	2889	3.66	1.2	3.59	3.1	4.1	8.7	1.01

SD: Standard deviation; CO: Carbon monoxide; NO: Nitrogen monoxide; NO_2: Nitrogen dioxide; NO_x: Nitrogen oxides; O_3: Ozone; SO_2: Sulfur dioxide; PM_{10}: coarse particulate matter; $PM_{2.5}$: fine particulate matter

Table S3. Pearson product-moment correlations to measure the relationships between air pollutants

	Temperature	Relative Humidity	Rainfall	PM_{10}	$PM_{2.5}$	CO	NO	NO_2	NO_x	O_3	SO_2
Temperature	1.00	<0.0001	<0.0001	<0.0001	<0.0001	<0.0001	<0.0001	<0.0001	<0.0001	<0.0001	<0.0001
Relative humidity	-0.14	1.00	<0.0001	<0.0001	<0.0001	<0.0001	<0.0001	<0.0001	<0.0001	<0.0001	<0.0001
Rainfall	-0.35	-0.19	1.00	<0.0001	<0.0001	<0.0001	<0.0001	<0.0001	<0.0001	<0.0001	<0.0001
PM_{10}	0.21	-0.35	0.13	1.00	<0.0001	<0.0001	0.2792	<0.0001	<0.0001	<0.0001	<0.0001
$PM_{2.5}$	0.31	-0.34	0.08	0.66	1.00	<0.0001	<0.0001	<0.0001	<0.0001	<0.0001	<0.0001
CO	-0.30	-0.29	0.38	0.20	0.16	1.00	<0.0001	<0.0001	<0.0001	<0.0001	<0.0001
NO	-0.49	-0.09	0.30	-0.02	-0.13	0.89	1.00	<0.0001	<0.0001	<0.0001	<0.0001
NO_2	-0.17	-0.42	0.33	0.41	0.42	0.89	0.71	1.00	<0.0001	<0.0001	<0.0001
NO_x	-0.32	-0.31	0.34	0.25	0.20	0.96	0.89	0.95	1.00	<0.0001	<0.0001
O_3	0.34	0.37	-0.44	-0.28	-0.11	-0.74	-0.67	-0.79	-0.80	1.00	<0.0001
SO_2	0.12	-0.31	0.21	0.51	0.51	0.64	0.41	0.79	0.68	-0.52	1.00

PM_{10}: coarse particulate matter; $PM_{2.5}$: fine particulate matter; CO: Carbon monoxide; NO: Nitrogen monoxide; NO_2: Nitrogen dioxide; NOx: Nitrogen oxides; O_3: Ozone; SO_2: Sulfur dioxide;

Table S4. Multinomial logistic regression analysis

	Obstructive vs Normal		Restrictive vs Normal		Mixed vs Normal	
	Crude OR (95% CI)	P	Crude OR (95% CI)	P	Crude OR (95% CI)	P
Age	1.01 (1–1.02)	0.0229	1.03 (1.01–1.04)	0.0005	1.06 (1.03–1.08)	<0.0001
Age groups (years)						
30–39	1.00		1.00		1.00	
40–49	1.04 (0.81–1.34)	0.7389	1.19 (0.71–2.00)	0.5109	0.72 (0.34–1.54)	0.4017
40–59	1.16 (0.91–1.48)	0.2240	1.41 (0.86–2.31)	0.1696	1.30 (0.68–2.48)	0.4301
≥60	1.38 (1.06–1.80)	0.0151	1.99 (1.20–3.31)	0.0079	3.78 (2.08–6.89)	<0.0001
Gender						
Male	1.00		1.00		1.00	
Female	1.15 (0.97–1.37)	0.1020	1.21 (0.87–1.68)	0.2699	1.92 (1.25–2.95)	0.0029
Anthropometric parameters						
Height	0.99 (0.98–1.00)	0.1116	0.96 (0.94–0.98)	<0.0001	0.92 (0.90–0.95)	<0.0001
Weight	1.00 (0.99–1.00)	0.4364	0.99 (0.97–1.00)	0.0856	0.95(0.94–0.97)	<0.0001
Body Adiposity Index	1.02 (1.00–1.05)	0.0368	1.08 (1.04–1.12)	0.0002	1.08 (1.03–1.14)	0.0009
Body Roundness Index	1.03 (0.96–1.11)	0.4228	1.21 (1.06–1.38)	0.0054	1.00 (0.83–1.20)	0.9863
Biochemical data						
Hematocrit	0.98 (0.96–0.99)	0.0227	0.99 (0.96–1.03)	0.6305	0.94 (0.90–0.99)	0.0096
Glycohemoglobin	1.20 (1.08–1.33)	0.0008	1.38 (1.19–1.61)	<0.0001	1.17 (0.92–1.48)	0.2038
Albumin	0.94 (0.66–1.34)	0.7304	0.51 (0.25–1.01)	0.0539	0.34 (0.15–0.79)	0.0122
Creatinine	0.80 (0.58–1.10)	0.1754	0.41 (0.17–0.99)	0.0466	0.10 (0.03–0.34)	0.0002
Comorbidities						
Diabetes mellitus type 2	1.48 (1.01–2.17)	0.0461	2.00 (1.06–3.77)	0.0312	1.51 (0.64–3.56)	0.3433
Monitoring region						
Northern region	1.00		1.00		1.00	
Central region	0.61 (0.47–0.80)	0.0003	0.47 (0.27–0.82)	0.0075	0.16 (0.07–0.34)	<0.0001
Southern region	0.59 (0.48–0.73)	<0.0001	0.70 (0.47–1.05)	0.0829	0.32 (0.21–0.50)	<0.0001
Meteorological factors						
Temperature	1.19 (1.07–1.34)	0.0022	1.10 (0.89–1.37)	0.3726	0.83 (0.65–1.06)	0.1285
Relative humidity	1.05 (1.02–1.09)	0.0029	1.00 (0.94–1.07)	0.8937	1.06 (0.98–1.15)	0.1332
Rainfall	0.08 (0.01–0.44)	0.0038	16.15 (0.64–404.91)	0.0905	241.56 (4.81–12134.04)	0.0060
Air pollutants						
PM_{10}	0.99 (0.98–0.99)	<0.0001	1.00 (0.99–1.01)	0.6971	0.99 (0.98–1.00)	0.0742
$PM_{2.5}$	0.98 (0.97–0.99)	<0.0001	1.00 (0.98–1.01)	0.9357	0.97 (0.95–0.98)	0.0002
CO	1.29 (0.80–2.07)	0.3019	3.21 (1.44–7.18)	0.0044	9.11 (3.90–21.3)	<0.0001
NO	1.02 (0.99–1.04)	0.0686	1.03 (0.99–1.07)	0.1501	1.09 (1.06–1.13)	<0.0001
NO_2	1.00 (0.98–1.01)	0.5917	1.03 (0.99–1.06)	0.0642	1.07 (1.03–1.11)	0.0005
NO_X	1.00 (0.99–1.01)	0.6450	1.02 (0.99–1.03)	0.0709	1.04 (1.03–1.06)	<0.0001
O_3	1.01 (0.98–1.03)	0.5472	0.98 (0.94–1.03)	0.4648	0.91 (0.86–0.96)	0.0004
SO_2	1.07 (0.99–1.14)	0.0820	1.16 (1.01–1.33)	0.0318	1.30 (1.11–1.52)	0.0013

PM_{10}: coarse particulate matter; $PM_{2.5}$: fine particulate matter; CO: Carbon monoxide; NO: Nitrogen monoxide; NO_2: Nitrogen dioxide; NO_x: Nitrogen oxides; O_3: Ozone; SO_2: Sulfur dioxide

Table S5. Interaction terms (cross-product terms)

Interaction terms	Obstructive vs Normal β (SE)	P	Restrictive vs Normal β (SE)	P	Mixed vs Normal β (SE)	P
Temperature by monitoring region	0.82 (0.14)	<0.0001	1.38 (0.26)	<0.0001	1.66(0.32)	<0.0001
Meteorological by air pollution factor						
Temperature by PM_{10}	0.01 (0.00)	0.0045	0.00 (0.01)	0.7818	0.01 (0.01)	0.4108
Temperature by $PM_{2.5}$	0.02 (0.01)	0.0065	0.03 (0.01)	0.0062	0.06 (0.01)	0.0002
Temperature by CO	-0.66 (0.28)	0.0191	0.34 (0.53)	0.5176	-0.93 (0.67)	0.1634
Temperature by NO	-0.02 (0.02)	0.2386	-0.08 (0.04)	0.0200	-0.05 (0.04)	0.2079
Temperature by NO_2	-0.02 (0.01)	0.0195	-0.03 (0.02)	0.1320	-0.06 (0.02)	0.0102
Temperature by NO_X	-0.02 (0.01)	0.0032	-0.02 (0.01)	0.1236	-0.03 (0.01)	0.0384
Temperature by O_3	0.04 (0.02)	0.0085	0.07 (0.03)	0.0144	0.11 (0.03)	0.0012
Temperature by SO_2	0.04 (0.04)	0.3124	-0.11 (0.09)	0.1964	-0.12 (0.10)	0.2269
Monitoring region by air pollution factor						
Monitoring region by PM_{10}	0.00 (0.00)	0.9475	-0.02 (0.01)	0.0103	0.00 (0.01)	0.8603
Monitoring region by $PM_{2.5}$	0.00 (0.01)	0.7723	-0.01 (0.01)	0.5219	0.00 (0.01)	0.8597
Monitoring region by CO	1.33 (0.37)	0.0003	1.64 (0.64)	0.0107	1.88 (0.82)	0.0219
Monitoring region by NO	0.07 (0.03)	0.0129	-0.11 (0.06)	0.0391	0.02 (0.07)	0.7354
Monitoring region by NO_2	0.04 (0.01)	<0.0001	0.01 (0.02)	0.5828	0.06 (0.02)	0.0128
Monitoring region by NO_X	0.03 (0.01)	0.0002	0.00 (0.01)	0.9749	0.04 (0.02)	0.0301
Monitoring region by O_3	0.00 (0.01)	0.7198	0.04 (0.02)	0.1020	0.02 (0.03)	0.4392
Monitoring region by SO_2	0.24 (0.04)	<0.0001	0.02 (0.08)	0.8167	0.17 (0.08)	0.0314

SE: Standard error; CO: Carbon monoxide; NO: Nitrogen monoxide; NO_2: Nitrogen dioxide; NO_x: Nitrogen oxides; O_3: Ozone; SO_2: Sulfur dioxide; PM_{10}: coarse particulate matter; $PM_{2.5}$: fine particulate matter

Table S6. Effect modification (interaction) associations between monitoring regions and temperature

	Monitoring region					
	Northern	Central	Southern	Chi Square	P	Cramer V
Temperature				682.2606	<0.0001	0.4860
<24.33 ℃	327 (57.3)	494 (88.5)	482 (27.4)			
≥24.33 ℃	244 (42.7)	64 (11.5)	1278 (72.6)			

Temperature groups were classified as being lower (<24.33 ℃) and higher (≥ 24.33 ℃) according to the mean temperature.

Effect size statistic	Values	Interpretation of effect size
Cramer’s V for nominal data	.00 and under .10	Negligible association
	.10 and under .20	Weak association
	.20 and under .40	Moderate association
	.40 and under .60	Relatively strong association
	.60 and under .80	Strong association
	.80 and under 1.00	Very strong association

Cramer’s V is commonly used to describe the magnitude of association between categorical variables for a contingency table larger than 2 x 2.

Table S7. Descriptive statistics of air pollutants divided by turning point temperature in the four lung function groups and monitoring regions in the four lung function groups

	Normal Spirometry	Obstructive Impairment	Restrictive Impairment	Mixed Impairment	P value
n	1902	733	154	100	
Temperature categories					
PM_{10} and $PM_{2.5}$					
<24.3 (℃)	851 (44.7)	304 (41.5)	65 (42.2)	43 (43.0)	
≥24.3 (℃)	1051 (55.3)	429 (58.5)	89 (57.8)	57 (57.0)	0.4797
CO, NO, NO_2, NO_X, and SO_2					
<24.6 (℃)	1283 (67.5)	412 (56.2)	85 (55.2)	57 (57.0)	
≥24.6 (℃)	619 (32.5)	321 (43.8)	69 (44.8)	43 (43.0)	<0.0001
O_3					
<24.9 (℃)	1546 (81.3)	526 (71.8)	107 (69.5)	72 (72.0)	
≥24.9 (℃)	356 (18.7)	207 (28.2)	47 (30.5)	28 (28.0)	<0.0001
Monitoring area					
Northern and Central Regions	702 (36.9)	321 (43.8)	57 (37.0)	49 (49.0)	
Southern Region	1200 (63.1)	412 (56.2)	97 (63.0)	51 (51.0)	0.0018

PM_{10}: coarse particulate matter; $PM_{2.5}$: fine particulate matter; CO: Carbon monoxide; NO: Nitrogen monoxide; NO_2: Nitrogen dioxide; NO_x: Nitrogen oxides; O_3: Ozone; SO_2: Sulfur dioxide
